# Global Health Education Amidst COVID-19: Disruptions and Opportunities

**DOI:** 10.5334/aogh.3088

**Published:** 2021-02-02

**Authors:** Stevan Weine, Maarten Bosland, Chandrika Rao, Marcia Edison, Daniel Ansong, Stacey Chamberlain, Agnes Binagwaho

**Affiliations:** 1 University of Illinois at Chicago Department of Psychiatry and Center for Global Health, USA; 2 University of Illinois at Chicago, Department of Pathology and Center for Global Health, USA; 3 Ramaiah Medical College, Bangalore, India; 4 University of Illinois at Chicago, Department of Medical Education and Center for Global Health, USA; 5 School of Medicine & Dentistry, School of Medical Sciences, Kwame Nkrumah University of Science and Technology, Ghana; 6 University of Illinois at Chicago, Department of Emergency Medicine; and Center for Global Health, USA; 7 University of Global Health Equity, Rwanda

## Abstract

This viewpoint examines the impact of COVID-19 travel bans and remote education on the global health education of students from high-income countries (HIC) and low- and middle-income countries (LMIC) and explores potential opportunities for strengthening global health education based upon more dispersed and equitable practices. Global health is unique in the opportunities it can offer to students during the pandemic if programs can manage and learn from the pandemic’s many challenges. Global health educators can: shift to sustainable remote engagement and mobilize resources globally to facilitate this; collaborate with partners to support the efforts to deal with the current pandemic and to prepare for its next phases; partner in new ways with health care professional students and faculty from other countries; collaborate in research with partners in studies of pandemic related health disparities in any country; and document and examine the impact of the pandemic on health care workers and students in different global contexts. These strategies can help work around pandemic travel restrictions, overcome the limitations of existing inequitable models of engagement, and better position global health education and face future challenges while providing the needed support to LMIC partners to participate more equally.

The COVID-19 pandemic has profoundly disrupted higher education across the world, in both high-income countries (HIC) and low- and middle-income countries (LMIC) [1,2,3]. Among all the higher education fields impacted, global health may be unique in the opportunities it can offer to students during the pandemic if programs can manage and learn from the pandemic’s many challenges.

Global health education programs are a relative newcomer to higher education across the globe and have expanded considerably over the past two decades, especially in the U.S. [4]. Today, global health students in schools of medicine, public health, nursing, and others are impacted by the pandemic in all the same ways as their schoolmates in other programs, but what uniquely disrupted their education has been the current inability to travel, especially for students from the global North.

Global health education often includes a field experience organized with partners locally or internationally, as well as interactions with visiting faculty and students from different global health settings, in most cases from HICs to LMICs, for educational and research collaborations [5]. These experiences add value to what can be learned from lectures, readings, and classroom discussion. The prolonged suspension of these experiences presents serious challenges to global health education as it has been practiced.

At the beginning of the pandemic in March 2020, most U.S. universities imposed across-the-board travel bans for faculty and students, and many LMICs countries, such as in Africa, imposed the same for everybody by closing their airports [6,7]. They presumably did so to prevent a returning traveler spreading the infection to non-travelers. To date the pandemic rages on globally, especially in the U.S. which has had more infections than any LMIC [8]. Most countries do not currently allow U.S. visitors because of the risks those travelers pose to their own populations [9].

This is not the first time global health education travel has been disrupted. Travel was temporarily shut down in many countries after the eruption of the volcano Eyjafjallajökull, just after 9/11 [10], proximate to other terrorist attacks and wars, and the West Africa Ebola outbreak of 2013–2016 [11], but never on this global scale and for this duration.

In prior situations, efforts were made by global health educators to engage students in education, volunteerism, or scholarship related to the very causes of disruption. However, the challenges of responding to the interruptions brought on by this pandemic have been made more complicated by several factors.

The disruptions to education due to the COVID-19 pandemic have been more profound compared to those caused by other global crises. In March 2020, health care professions’ students in many countries, including the U.S., were temporarily removed from clinical clerkships [12,13]. This response was justified by concerns over the safety of students, faculty, and patients (e.g., because of lack of sufficient personal protective equipment). In some instances, this could have also reflected a limited capacity to adequately provide medical education in the face of the pandemic (e.g., due to overwhelming clinical duties or to inexperience with virtual education) [14].

In summer 2020, the health risks related to COVID-19 were deemed more manageable, and most students returned to their clerkships in the U.S. [3,15]. However, students, faculty, and visitors at many universities are still unable to travel internationally, and that does not appear likely to change for the foreseeable future.

Another challenge is that COVID-19 has brought forward major persistent health inequities associated with race and ethnicity in the U.S. and other countries [16]. The deaths of George Floyd and many others and the Black Lives Matter protests have accelerated an overdue reckoning in global health regarding racism and colonialism [17]. Global health has a history of being rooted in tropical medicine, which was initially used to support Western colonialism and continues to underlie inequities in global politics, economics, education, and health care [18].

In light of these developments, the existing practice of U.S. students travelling abroad, previously critiqued for often being short-term, extractive, and excessively expensive, becomes even less defensible [19]. Although equity-focused best practice guidelines have been articulated, it has been difficult for U.S. global health programs to develop feasible models in which students and faculty from LMICs could have parallel exchanges involving travel to the U.S. and other HICs [4].

Given the marked challenges of the present pandemic context, what can global health educators do to support the education of their students who are not able to travel abroad or have contact with visitors from abroad? No less important, what can be done to support students in LMICs whose capacity for remote education can be impacted by limitations of Wi-Fi and internet bandwidth and electrical power, as well as internet and equipment cost obstacles?

These are profoundly challenging questions. We believe global health educators and students can take steps to both enhance educational opportunities for HIC and LMIC students and to address some of the innumerable challenges of the pandemic to health and society in all countries. In particular, the pandemic raises many important issues related to disparities, inequity, and neocolonialism, which are central to the very definition of global health [20]. For example, among racial and ethnic minorities in the U.S., COVID-19 is more highly prevalent and lethal, economic pressures are higher, and access to a potential vaccine may be less compared to white and affluent populations [21]. In LMICs, access to testing, adequate treatments (e.g., oxygen), and vaccines is overall far less than in HICs, and there are often great in-country inequities. For those very reasons, the pandemic presents opportunities for developing new approaches to global health education which depart from inequitable practices rooted in global health’s colonial past [18].

Here are several practical suggestions for what educators can do, some of which are already operational:

1. **Shift programs so students can engage remotely and regularly with academic, clinical, and community programs in different global health contexts.** Dispersion practices via digital platforms which enable businesses and universities from HICs to maintain direct relationships with their clients and students during the pandemic can be used to sustain global health partnerships including students’ activities [22]. By widely using available communication platforms such as Zoom and WebEx, more popular in the U.S., and those more popular abroad, including WhatsApp and Moodle and remote learning platforms such as Lecturio and ScholarX, students from universities in HICs and LMICs can engage with partners in other countries and establish new partnerships, with potentially far greater continuity of involvement than short-term travels allows. Although simply engaging online may be too obvious to deserve mentioning, changing the global health education paradigm from centering on periodic trips to continuous on-line engagement could be a profound transformation. For example, virtual didactics, trainings, and fellowships could be offered to those in both HICs and LMICs. In support of this development and mutual learning, LMICs and HICs need to engage in global mobilization for the provision of resources including, but not limited to, computers and internet access needed in LMICs.
2. **Form student-to-student partnerships between health care professional students in LMICs and HICs to conduct equitable joint projects.** Some of these students could be open to joint bi-directional learning opportunities, sometimes referred to as twinning [23], although there is a need to address obstacles due to Wi-Fi and internet bandwidth and electric power limitations, and the cost of computers and mobile data. One example is a collaboration between students in the University of Illinois at Chicago Global Medicine program and the Federation of African Medical Students’ Associations who are jointly developing an online mental health and psychosocial support intervention. Industry, for example pharmaceutical firms, could further the partnerships between student and healthcare professionals by providing students in both LMICs and HICs with support or opportunities for practical experiences.
3. **Collaboratively develop training and best practice materials that can support LMIC and HIC partners as they cope with the pandemic’s current and future challenges.** Students and faculty in LMICs and HICs can collaborate in developing materials that can facilitate the training and best practices of medical and public health practitioners and policymakers in the face of the evolving pandemic. These materials can address COVID-19 relevant topics including vaccine distribution, clinical care management, workforce enhancement, disease surveillance and reporting, and psychosocial coping. For example, one such effort could entail developing public health education materials for primary and secondary schools on the coming vaccine, contributing to the fight against vaccine hesitancy.
4. **Engage in collaborative studies of pandemic-related global health disparities, especially related to race, ethnicity, and refugee and migrant populations.** There is an urgent need to conduct collaborative research in these areas of disparities, presenting students in global health education programs worldwide with great opportunities to advance knowledge in ways that can speak to practice and policy. One example of a collaboration could be a mixed-methods study of the COVID-19 experiences of migrant day laborers in both HICs and LMICs that would result in recommendations for action.
5. **Document and examine involvement of health care workers and students in responding to the pandemic in different countries.** The stories of health care workers’ roles, strengths, vulnerabilities, and coping measures in response to the pandemic in many different contexts need to be told and understood. This also applies to COVID’s impact on the education of students in HICs and LMICs and their participation in volunteerism. The knowledge gained from understanding the struggles and resilience of health professionals and students can inform future educational programs in many countries. For example, global health students could collect oral histories, conduct ethnographic studies of health care workers, or teach students to write opinion pieces.

Additionally, while global health educators in HICs are working to create opportunities for their students at their home institutions, they can contribute to the global health field in several ways. Educators can share best practices such as how to use distance technology or how to maintain mentorship networks across countries. Educators can also conduct evaluations that measure the consequences of COVID-19 and assess and compare the impact of innovative educational programs in HICs and LMICs.

Most importantly, global health educators should not see COVID-19 as merely a temporary inconvenience but rather as an opportunity for them and their students – the future leaders of global health – to get smarter, stronger, and better prepared for adversities which the future may hold. This is not the first pandemic or disaster to disrupt global health education, and it will certainly not be the last. This pandemic may give us a new view of what is needed to establish or sustain the future global health workforce, which education programs can be addressing.

Global health education also needs to help its students to be more equitably engaged. Thus, the pandemic presents an opportunity for de-colonizing global health education by overcoming its dependence upon short-term travel by student from HICs. Instead of relying on inequitable models of engagement with LMICs, that are dependent on often prohibitively high travel costs, global health educators can devise more bi-directional educational practices that embody the value of equity and do not amplify disparities and neocolonialism.

In conclusion, this is a time for openness, flexibility, innovation, and entrepreneurialism so as to develop novel models of partnership and educational engagement which can open a new and more equitable chapter in global health education.
